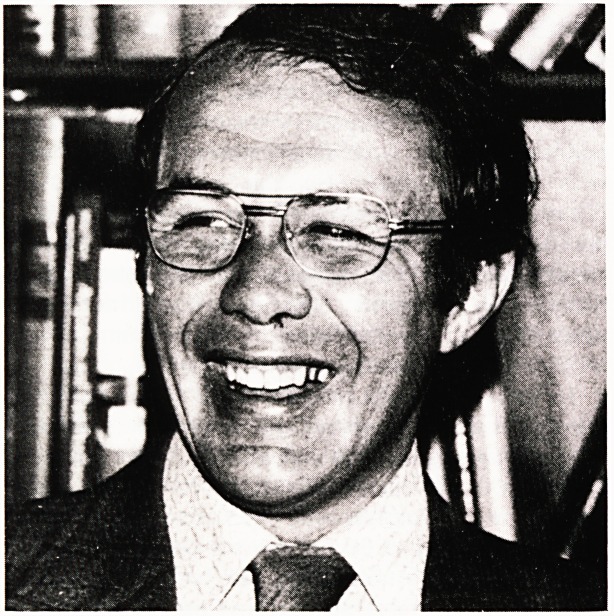# W. G. J. Hampson

**Published:** 1979

**Authors:** 


### Obituary

### W. G. J. Hampson
### M.A., B.M., B.Ch., F.R.C.S.Ed.

Mr. W. G. J. Hampson, Consultant Orthopaedic Surgeon at Southmead and Winford Orthopaedic Hospitals died on 27th December 1978 at the age of 41.

William Grenville Johnson Hampson was born in Cheshire on 9th December 1937 and was educated at Lady Manners School Bakewell, Corpus Christi College, Oxford, and St. Mary's Hospital. He graduated B.A. (Physiology) in 1960 and M.A., B.M., B.Ch., in 1964. After qualification his first appointment was to the orthopaedic unit at St. Mary's and this post produced a life-long enthusiasm for the speciality. Further posts at St. Mary's followed and he worked at Coventry, Oxford, Liverpool and Edinburgh while preparing for the fellowship. He was registrar at Harlow Wood Orthopaedic Hospital before moving to Bristol as Senior Registrar in Orthopaedics at the Royal Infirmary and Winford Orthopaedic Hospital in 1971. Here his surgical skills blossomed and he began to develop the research interests that occupied an important part of his further professional career. He organized a study into the management of post-operative venous thrombosis which received wide recognition as a major contribution in this field. In 1973 he was awarded a British Orthopaedic Association travelling scholarship to study biomechanics in Sweden, and subsequently it became one of his central ambitions to promote the setting up of a biomechanics centre in Bristol. While still a Senior Registrar he was awarded a major research grant from the Arthritis and Rheumatism Council to investigate the pathology of back pain. In 1974 he was elected a member of the British Orthopaedic Research Society. He became Consultant at Southmead and Winford Orthopaedic Hospitals in 1975 with responsibility also for the Accident and Emergency services at Southmead. The next year he was awarded one of the coveted British Orthopaedic Association ABC fellowships to visit and lecture at leading orthopaedic centres in the United States and Canada.

This considerable research activity was combined with surgical skills of a high order, and also with great ability as a clinician. Probably his most outstanding attribute was his ability to get on with people, at all levels in his professional and personal life. His patients were devoted to him, he had an unusually wide circle of close friends, and even his research to a large extent depended on this ability to create close friendly relationships with workers in other fields, such as veterinary medicine, medical physics and rheumatology.

He had wide interests outside medicine. In his youth he played rugger, rowed and climbed with some distinction and in later years pursued such diverse interests as sailing, photography, vintage cars, gardening, owl breeding and the love of good wines.

The last two months of his life will never be forgotten by the many colleagues and friends who visited him. The end was inevitable and painful but Gary faced this with his characteristic courage and humour, giving strength to his family and friends until the last.

Our deepest sympathy goes to his wife Elizabeth and his young son Matthew.

## Figures and Tables

**Figure f1:**